# Evaluation of gantry rotation overrun in axial CT scanning

**DOI:** 10.1120/jacmp.v15i5.4901

**Published:** 2014-09-08

**Authors:** Atsushi Fukuda, Pei‐Jan P. Lin, Kosuke Matsubara, Tosiaki Miyati

**Affiliations:** ^1^ Department of Radiology Shiga Medical Center for Children Moriyama Shiga Japan; ^2^ Division of Health Sciences Graduate School of Medical Sciences, Kanazawa University Kanazawa Ishikawa Japan; ^3^ Department of Radiology Virginia Commonwealth University Medical Center Richmond VA USA

**Keywords:** CT scanner, gantry overrun, gantry rotation time, solid‐state detector, CTDI

## Abstract

The purpose of this study was to develop and evaluate a simple method to assess gantry rotation overrun in a single axial CT scanning. The exposure time in the axial scanning was measured at selected nominal rotation times (400, 700, and 1000 ms) using a solid‐state detector, the RTI's CT dose profiler (CTDP). CTDP was placed at the isocenter and the radiation dose rate signal (profile) was recorded. Subsequently, the full width of this profile was determined as the exposure time ($T_{\text{axial}}$). Next, CTDP was positioned on the inner cover of the gantry with a sheet of lead (1 mm thick) placed on top of the detector. Gantry rotation time ($T_{\text{helical}}$) was determined by the time between two successive radiation peaks during continuous helical scanning. The gantry overrun time ($T_{\text{overrun}}$) is, thus, determined as $T_{\text{axial}}‐T_{\text{helical}}$. The exposure times in the axial scanning, $T_{\text{axial}}$, obtained with CTDP for nominal rotation times of 400, 700, and 1000 ms were 409.5, 709.6, and 1008.7 ms, respectively. On the other hand, the measured gantry rotation times, $T_{\text{helical}}$, were 400.0, 700.3, and 999.8 ms, respectively. Therefore, the overruns were 9.5, 9.3, and 8.9 ms for nominal rotation times of 400, 700, and 1000 ms, respectively. The evaluation of overrun in axial scanning can be accomplished with the measurements of both the exposure time in axial scanning and the gantry rotation time. It is also noteworthy that in this context, overrun implies overexposure in axial scanning, which is still used, particularly, in head CT examination.

PACS number: 87.57.Q‐

## I. INTRODUCTION

CT dose index (CTDI) is a widely accepted standard metric to quantify the radiation output from CT examinations.^1^, ^2^ By definition, the ${\text{CTDI}}_{100}$ is the average dose imparted by a single axial scanning using a standard 100 mm pencil chamber dosimeter inside a polymethyl methacrylate (PMMA) phantom.

With the advent of helical scanning and cone‐beam CT technology, the definition and its measurement conditions of ${\text{CTDI}}_{100}$ dosimetry would not reflect appropriately the geometry of the scan environment. This is because the 100 mm pencil chamber dosimeter is simply too short to detect contributions from the primary and scattered radiation beyond the chamber range along z‐axis.^(3)^ American Association of Physicists in Medicine (AAPM) published a report of comprehensive methodology for the evaluation of radiation dose in CT, and suggested measurement of “dose equilibrium” in place of CTDI which can be used for axial, helical, and cone‐beam scanning with or without table translation.^(4)^

While the trend is to employ helical scanning for most CT examinations, use of the axial scanning for routine head CT examinations^(5)^ and CTDI dosimetry measurement are conducted under axial scanning, albeit AAPM provided dose equilibrium for the evaluation of radiation dose in CT. Additionally, cone‐beam CT technology was developed and employed to perform coronary CT angiography without table translation.^(6)^ The axial and cone‐beam scanning require X‐ray tube startup and power down time (overrun), which extend the time duration of X‐ray exposure.

Gantry overrun can be calculated from the difference between the exposure time in axial scanning (or cone‐beam scanning, which can be considered as a type of axial scan without table translation) and gantry rotation time. The solid‐state detector was recently developed to record the radiation dose profile, and it could be applied to measure the exposure time and time‐varying average point dose rate.^(7)^ In contrast, a simple technique was recently described to measure gantry rotation time with two different types of solid‐state detectors.^(8)^ The aim of this study was to provide a simple approach to assess gantry overrun time in axial scanning.

## II. MATERIALS AND METHODS

### A. CT scanner and solid‐state detector system

The MDCT scanner (LightSpeed VCT, GE Healthcare, Milwaukee, WI) employed for this investigation was equipped with 64 rows of detector array. The scan parameters employed were: tube potential of 120 kVp, tube current of 100 mA, and total collimation width of 40.0mm(0.625mm×64rows).

The solid‐state detector system, which is the CT dose profiler (CTDP) detector, was connected to the Piranha electrometer (RTI Electronics, Mölndal, Sweden). The probe was specifically designed for CT dosimetry applications.^(7)^ Data collection and analysis were performed with the software “Ocean”, available from RTI. The collected signal was subsequently sent to a laptop through a USB cable. The laptop computer, running the “Ocean” software, captured the signal and displayed the radiation waveform for analysis.

### B. Measurement of the exposure time in single axial scanning

**Figure 1:**
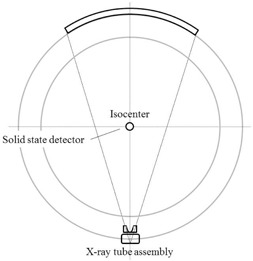
Experimental arrangement for measurement of exposure time in axial scanning. The detector was positioned free in‐air at the geometrical isocenter of the gantry for exposure time measurement under the axial scan mode of operation.

The CTDP probe is suspended free in‐air at the geometrical isocenter of the gantry, as shown in Fig. 1. After aligning the probe, the radiation dose profile is measured under the axial scan mode, with nominal rotation times of 400, 700, and 1000 ms. The output signal from CTDP is fed into the Piranha electrometer and the laptop. The data obtained can be exported to Microsoft Excel spreadsheet program for further analysis, manipulation, and processing. The exposure time ($T_{\text{axial}}$) is then determined by measuring the full width of the radiation dose profile.

To avoid the modulation and attenuation of the radiation intensity due to the tabletop, care should be taken to ensure the tabletop is located outside of the imaging plane at all times while the radiation dose profile is collected.

### C. Measurement of the gantry rotation time

**Figure 2:**
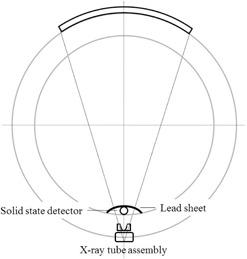
Experimental arrangement for measurement of gantry rotation time. The detector was positioned on the inner bottom of the gantry cover for gantry rotation time measurement under the helical scan mode of operation. A lead sheet was placed on top of the detector to reduce the effect of unwanted primary radiation.

The CTDP probe is positioned on the gantry cover, as shown in Fig. 2. Notice that to shield any stray radiation from reaching the detector, a sheet of lead (1 mm thick) is placed on top of the detector. Thus, the detector is exposed the primary radiation only when the X‐ray tube passes by the detector at the bottom of the gantry.

The CT scanner is operated under the helical scan mode with nominal rotation times of 400, 700, and 1000 ms, and the examination table may be in the imaging plane because its presence has no effect on the primary beam entering the detector. A peak of radiation signal is observed when the X‐ray tube briefly passes by the detector. Therefore, the time duration between two successive peaks of radiation signal is the gantry rotation time ($T_{\text{helical}}$).

## III. RESULTS

**Figure 3:**
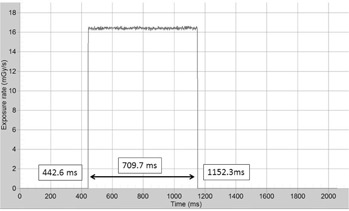
Illustration of the detector output signal using an RTI's CTDP probe for measurement of the exposure time in axial scanning. The CTDP probe was positioned free in‐air at the geometrical isocenter of the gantry, and the exposure time measurement was conducted with a nominal gantry rotation time of 700 ms. The exposure time determined from the full width of this profile was 709.7 ms.

Depicted in Fig. 3 is the radiation dose profile from the CTDP probe positioned at the isocenter in the axial scanning mode for a nominal rotation time of 700 ms. Using the cursor provided in the “Ocean” software, the exposure time determined from the full width of the radiation profile is 709.7 ms.

**Figure 4:**
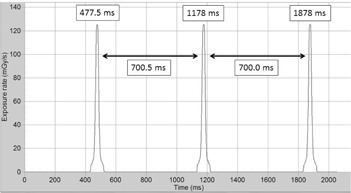
Illustration of the detector output signals/peaks using an RTI's CTDP probe for measurement of the gantry rotation time. The CTDP was positioned on the inner bottom of the gantry cover with a 700 ms nominal gantry rotation time. The three peaks were registered at 477.5, 1178, and 1878 ms, respectively. The measured average gantry rotation time was 700.3 ms.

The radiation dose profile when the CTDP probe was placed on the bottom of the gantry cover under helical scanning mode for a nominal rotation time of 700 ms is shown in Fig. 4. Any pair of two successive dose profiles (peaks) can be employed to determine the gantry rotation time ($T_{\text{helical}}$). There are three peaks located at rotation time of 477.5, 1178, and 1878 ms, respectively. Therefore, the average gantry rotation time is 700.3 ms.

Measurements were repeated three times for each nominal setting. Table 1 shows the mean and standard deviation of the exposure time, the measured gantry rotation time, gantry overrun time, and the excess percentage radiation dose. The excess percentage radiation dose is calculated as:

(1) $$D_{e}=(D_{\text{axial}}-D_{\text{one}})/D_{\text{one}}$$

where $D_{\text{axial}}$ and $D_{\text{one}}$ are integral doses during full width of this radiation profile and one rotation time, respectively. All gantry overruns and the excess percentage radiation doses were within 9.5 ms and 1.8%, respectively.

**Table 1:** The exposure times, measured gantry rotation times, gantry overruns, and the excess percentage radiation doses^a^

*Nominal Gantry Rotation Time (ms)*	*Exposure Time (ms)*	*Measured Gantry Rotation Time (ms)*	*Gantry Overrun (ms)*	*Excess Percentage Radiation Dose (%)*
400	409.5±0.1	400.0±0.2	9.5±0.2	1.8
700	709.6±0.1	700.3±0.4	9.3±0.4	1.0
1000	1008.7±0.1	999.8±0.2	8.9±0.2	0.7

a Errors are standard deviation.

## IV. DISCUSSION

Because of the continued growth in CT utilization in recent years, an increased emphasis has been placed on the importance of quality assurance/control and patient radiation dose assessment during CT examinations.^(9)^ To design appropriate scanning protocols, it is important to understand not only the variable settings, but also the methodology to assess accurate radiation dose.^1^, ^4^, ^10^

Previously, the standard method of measuring CT radiation output required a 100 mm pencil ionization chamber, the CTDI phantom, and a single axial scan to obtain ${\text{CTDI}}_{100}$ and ${\text{CTDI}}_{w}$.^(11)^ This technique was suitable for clinical environment because 1) ${\text{CTDI}}_{100}$ can be measured without a time‐consuming process, and 2) the technique does not require complicated QA tools or software. At present, ${\text{CTDI}}_{\text{vol}}$ is still used to account for helical scanning parameters in clinical setting. The “dose equilibrium” proposed by AAPM is yet to be accepted to replace the traditional CTDI dosimetry approach.^1^, ^4^ In this study, it is shown that the excess percentage radiation doses decreased as a function of (increasing) nominal gantry rotation time. Therefore, it is suggested that the overrun should be taken into account when the faster rotation time is employed in CTDI dosimetry.

Nonhelical scanning is still used not only for head CT,^(5)^ but also coronary CTA^(6)^ and brain perfusion study.^(12)^ Because these scanning require X‐ray tube startup and power down time (i.e., overrun), optimization of gantry overrun would be desirable to minimize the excess radiation to the patient.

In this investigation, a solid‐state detector, CTDP, was employed to measure both the exposure time in the axial scanning and gantry rotation time. The gantry overrun was then derived by subtracting the gantry rotation time from the exposure time in the axial scanning. The measurement methodology described herein should be of great assistance to physicists since the radiation detectors are readily available and require relatively short machine time to perform the measurements.

The gantry overrun time was 9.5 ms (2.4%) for a nominal gantry rotation time of 400 ms. As anticipated, the exposure time in axial scanning was longer than the nominal setting. This gantry overrun time of 9.5 ms is equivalent to 8.6° of gantry rotation, and the excess percentage radiation dose is 1.8%. Therefore, the gantry overrun should be minimized for further reduction of radiation dose in concert with the as‐low‐as‐reasonably‐achievable (ALARA) concept. This is especially important in the shorter gantry rotation time.

On the other hand, if the overrun is much longer than the 10 ms, the rotation time accuracy should be retuned. Furthermore, inclusion of the gantry overrun measurement should be considered when acceptance testing of a CT scanner is being planned.

Gantry rotation time measurements can be used not only to obtain the gantry overrun as shown in this study, but also to verify the error of table feed speed, which may result in image distortion along the z‐axis.^(13)^ While the gantry rotation time is an important scan parameter of any CT scanner, it was not explicitly included in the AAPM Reports 39^(14)^ and 83,^(15)^ nor in the American College of Radiology 2012 CT quality control manual.^(9)^ We believe verification of the gantry rotation time should be included in a comprehensive performance evaluation of a modern CT scanner.

Note that the present study has two limitations. The measurements were conducted at three nominal rotation time settings. It would be more desirable to evaluate the rotation times for other scanners at other time settings for completeness.

It was impossible to concurrently measure the gantry rotation time and the exposure time in axial scanning. As previously mentioned, the full width of radiation profile measurement requires single axial scanning, while the gantry rotation time measurement requires multiple (helical) scanning. Therefore, it was difficult to verify the actual gantry rotation time in axial scanning. However, we believe that the difference of the rotation times between the axial and helical scanning is insignificant because the same rotation time controller in CT scanner is applied.

## V. CONCLUSIONS

We proposed a simple method for the measurement of gantry overrun in modern commercial CT systems. We showed that measurement of gantry overrun can be accomplished with a solid‐state detector. In addition, although it is small, it is noteworthy that the measurement result of gantry overrun implies an overexposure to the patient. It is also suggested that evaluation of overrun should be included in a comprehensive acceptance testing of a CT scanner.

## Supporting information

Supplementary Material

Supplementary Material

Supplementary Material
