# Supplementary figures and images for: Evaluation of gantry rotation overrun in axial CT scanning
Source: J Appl Clin Med Phys. 2014 Sep 8;15(5):229–34. doi: 10.1120/jacmp.v15i5.4901 (PMC5711073; doi:10.1120/jacmp.v15i5.4901)

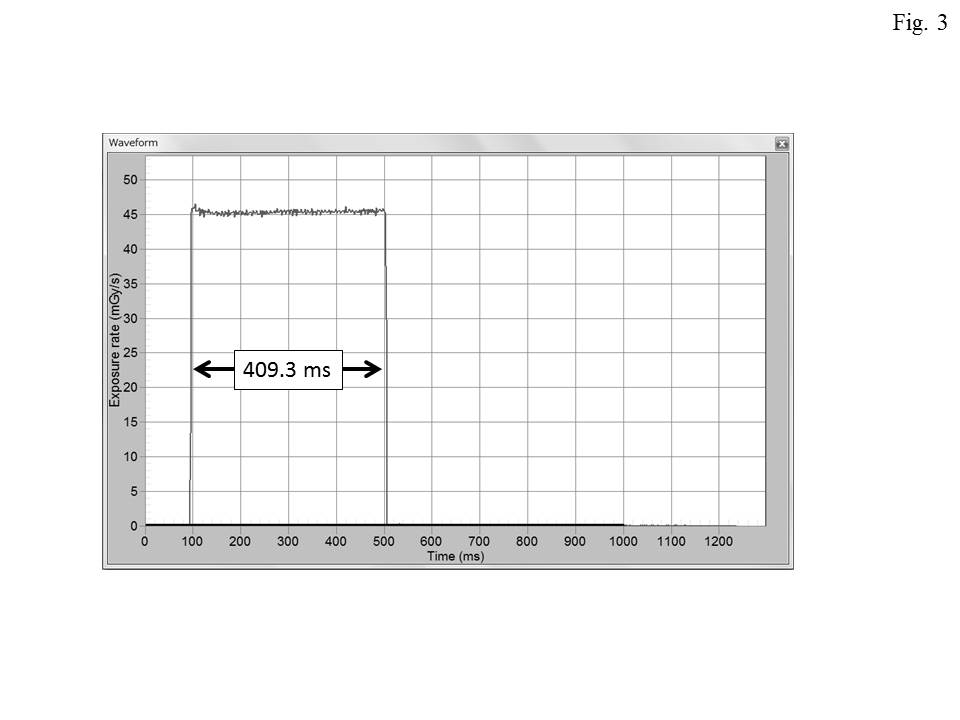

Supplement: Supplementary file 2 — Supplementary Material [file ACM2-15-229-s002.JPG]

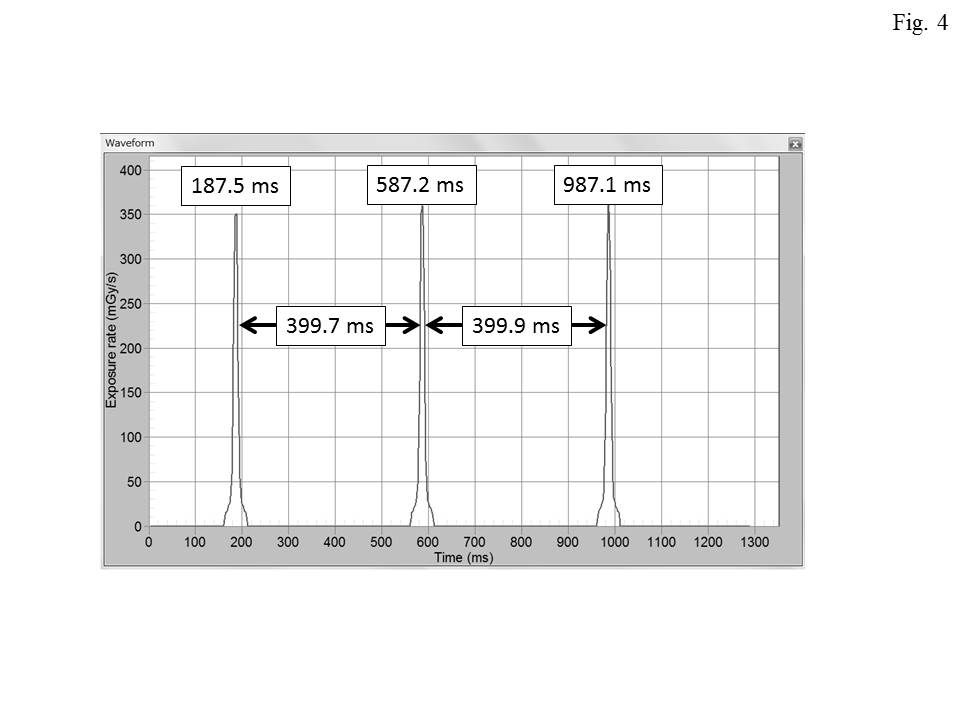

Supplement: Supplementary file 3 — Supplementary Material [file ACM2-15-229-s003.JPG]
